# Evaluation of Hemogram-Derived Inflammatory Markers in Healthy Cats

**DOI:** 10.3390/vetsci13030238

**Published:** 2026-03-01

**Authors:** Alper Erturk, Aliye Sagkan Ozturk, Ramazan Ozdemir

**Affiliations:** 1 Department of Internal Medicine, Veterinary Faculty, Hatay Mustafa Kemal University, Hatay 31040, Türkiye; asagkan@mku.edu.tr; 2 Department of Molecular Biochemistry and Genetic (Medicine), Institute of Health Sciences, Hatay Mustafa Kemal University, Hatay 31040, Türkiye; 3 Adana Metropolitan Municipality Stray Animal Shelter, Adana 01000, Türkiye; ramazan.ozdemir@adana.bel.tr

**Keywords:** feline, hematological inflammatory ratios, NLR, MLR, PLR, SII, AISI, SIRI

## Abstract

In the field of veterinary medicine, hematological indices such as the neutrophil-to-lymphocyte (NLR), monocyte-to-lymphocyte (MLR), platelet-to-lymphocyte (PLR), systemic immune–inflammatory index (SII), aggregate systemic inflammation index (AISI), and systemic inflammatory response index (SIRI) are increasingly recognized as cost-effective indicators of systemic inflammatory activity. Despite their diagnostic potential, standardized reference intervals for these metrics remain undefined in clinically healthy feline subjects. Consequently, the primary objective of this investigation was to establish baseline reference ranges for these inflammatory ratios in cats, thereby facilitating more accurate clinical interpretations and diagnostic assessments in feline practice. Clinical records were reviewed retrospectively, and cats showing no abnormalities on physical examination and presenting laboratory values within established reference ranges were included in the analysis. Following the sex-based analysis of 88 samples, the reference intervals were defined as follows: 0.8–7.1 for NLR; 0.1–0.6 for MLR; 22.7–374.6 for PLR, 130–2454 for SII, 39.5–1542.8 for AISI, and 0.2–6.1 for SIRI. Comparison by sex did not demonstrate statistically significant differences in any of the analyzed indices. These findings may offer a significant clinical framework for the interpretation of hematological inflammatory markers in feline patients.

## Simple Summary

This research focuses on establishing standard reference values for specific blood ratios in cats to improve diagnostic precision. Key markers like the neutrophil-to-lymphocyte (NLR), monocyte-to-lymphocyte (MLR), platelet-to-lymphocyte (PLR), systemic immune–inflammatory index (SII), aggregate systemic inflammation index (AISI), and systemic inflammatory response index (SIRI) are increasingly favored in veterinary medicine as they are easily derived from routine blood tests at no extra cost. These indices have proven essential for predicting outcomes in acute inflammatory, cancerous, hepatic, and gastrointestinal conditions, as well as serving as early indicators for heart and lung diseases. The study identified the following reference ranges: 0.8–7.1 for NLR; 0.1–0.6 for MLR; 22.7–374.6 for PLR; 130–2454 for SII; 39.5–1542.8 for AISI; and 0.2–6.1 for SIRI. These findings provide a practical clinical tool, allowing veterinarians to quickly compare patient data and make more accurate prognostic and predictive assessments across various diseases.

## 1. Introduction

Diagnostic approaches in small animal practice have increasingly incorporated newly identified biomarkers to enhance clinical assessment [1]. Within small animal veterinary practice, the complete blood count (CBC) is widely utilized as a standard diagnostic tool during routine examinations. In recent years, interest in novel inflammatory indices calculated from CBC parameters has increased, and the use of these biomarkers as readily available clinical tools has become widespread [2,3,4,5].

The neutrophil-to-lymphocyte ratio (NLR), monocyte-to-lymphocyte ratio (MLR), platelet-to-lymphocyte ratio (PLR), together with the systemic immune–inflammation index (SII), the aggregate index of systemic inflammation (AISI), and the systemic inflammatory response index (SIRI), are novel, popular, cost-effective, and readily accessible diagnostic and prognostic biomarkers, which associated with systemic inflammation since they are computed using routinely measured CBC parameters [6,7,8]. Compared with isolated hematological variables, these indices derived from CBC data may offer superior performance in evaluating systemic responses, as they provide a more accurate evaluation of immunological and inflammatory status by accounting for interactions among different blood cell types [9,10,11,12,13].

Investigations focusing on dogs have highlighted the potential prognostic relevance of NLR, MLR, and PLR in acute inflammatory diseases such as peritonitis, pancreatitis, and acute diarrhea [10,11,13,14,15,16], as well as in neoplastic conditions [17,18,19,20], and cardiovascular, intestinal, or hepatic pathologies [21,22,23,24,25,26]. Ratios among inflammatory markers, such as NLR, MLR, and PLR, have recently been investigated in cats with inflammatory and neoplastic diseases, and their potential clinical value has been explored for both diagnostic assessment and outcome prediction in this species [6,11,27,28,29].

SII, AISI, and SIRI have emerged as superior biomarkers within various pathological contexts in humans, most notably in cancer and cardiovascular disease [30,31,32]. When compared with traditional markers of inflammation, these three composite indices allow a more dependable assessment of systemic inflammatory activity and may serve as prognostic indicators [7,8]. However, their use in cats remains relatively limited, and only a few studies have evaluated the application of SII, AISI, and SIRI in this species [3,4,33,34,35]. The systemic immune–inflammation index (SII) is a composite biomarker that reflects the balance between immune system activity and inflammatory processes. The aggregate index of systemic inflammation (AISI) has emerged as a composite inflammatory indicator applied to evaluate disease related inflammatory burden in human medicine [36] and provides a holistic overview of inflammatory processes within the body [37,38]. The systemic inflammatory response index (SIRI) has been shown to be highly effective in predicting mortality outcomes in patients with a variety of medical conditions [37].

To date, there are no available data on hematological ratios and reference intervals derived from a large healthy cat population in which NLR, MLR, PLR, SII, AISI, and SIRI have been evaluated collectively. Most previous feline studies have focused on diseased populations, and the lack of standardized reference data derived from healthy cats limits the clinical interpretation of these indices. Without validated reference intervals, distinguishing physiological variation from early inflammatory alterations remains challenging in routine practice. Therefore, establishing population-based reference ranges is essential before these indices can be reliably integrated into feline clinical diagnostics. The aim of this retrospective study was to establish reference intervals for selected hemogram-derived inflammatory markers in a healthy cat population and to determine whether significant differences exist between sexes, thereby supporting their use in clinical practice.

## 2. Material and Methods

This retrospective study evaluated healthy cats using a retrospective review of medical records obtained from the database of the Adana Metropolitan Municipality Stray Animal Shelter between 2020 and 2023. All cats included in the study were stray cats housed at this shelter. Written permission was obtained from the administration of the Adana Metropolitan Municipality Stray Animal Shelter. Given the retrospective nature of the study, formal ethical approval was not required.

### 2.1. Study’s Description

Variables such as age and sex were included without selection bias in the study. Only mixed-breed cats aged between 1 and 6 years were included, representing the young adult population. Information on preventive care, such as antiparasitic treatment or vaccination status, was not included in the study because these data were not available for all patients. Cats with no abnormalities detected on comprehensive physical examination and with hematological parameters within reference limits were included in the study.

### 2.2. Inclusion/Exclusion Criteria

Cats were included in the study if they underwent a comprehensive physical examination, including assessment of rectal body temperature, mucous membranes, hydration status, lymph node palpation, capillary refill time, and measurement of heart and respiratory rates, as well as urinalysis (urine dipstick testing). In addition, inclusion required CBC analysis comprising total white blood cell (WBC), lymphocyte (LYM), monocyte (MON), neutrophil (NEU), red blood cell (RBC), mean corpuscular volume (MCV), mean corpuscular hemoglobin concentration (MCHC), red blood cell distribution width (RDW), hemoglobin (Hb), hematocrit (HCT), and platelet count (PLT), measured using an automated hematology analyzer (BC-30 Vet, Mindray, Shenzhen, China). Only cats with findings within established reference limits were included. Cats with a known history of systemic illness, ongoing medical treatment, or incomplete clinical data were also excluded from the study.

### 2.3. Hematology Analysis and Hematology-Derived Inflammatory Markers Ratio Calculation

Blood samples were collected from the cephalic vein of all enrolled cats under manual restraint without sedation. Sampling was performed during routine clinical evaluation. For hematological analysis, blood samples were collected into K_3_EDTA-containing tubes and CBC analyses were performed within 5–10 min of sample collection. Blood sampling was performed once per cat, and no repeat sampling was conducted. To ensure methodological consistency, all samples underwent evaluation within one reference laboratory using a single analytical device. CBC were determined using an automated hematology analyzer (BC-30 Vet, Mindray, China), and routine internal quality control procedures were performed in accordance with the manufacturer’s recommendations. The analyzer’s automated flagging system was reviewed for each sample, and only results without instrument flags indicating potential abnormalities were included in the final analysis. Composite indices derived from the CBC were calculated manually. The reference laboratory expressed all WBC and platelet measurements using uniform units (10^9^/L). The hematological indices NLR, MLR, PLR, SII, AISI, and SIRI were determined using absolute values obtained from the hemogram analyzer, as described below.

NLR, defined as the ratio of neutrophils to lymphocytes;
MLR, defined as the ratio of monocytes to lymphocytes;
PLR, defined as the ratio of platelets to lymphocytes;
SII, calculated as the product of neutrophil and platelet counts divided by the lymphocyte count;
AISI, calculated as the product of neutrophil, monocyte, and platelet counts divided by the lymphocyte count;
SIRI, calculated as the product of neutrophil and monocyte counts divided by the lymphocyte count [37,39,40].

### 2.4. Statistical Analysis

The study included 88 clinically healthy cats. Nonparametric methods were preferred in accordance with the guidelines of the Clinical and Laboratory Standards Institute (CLSI) and the International Federation of Clinical Chemistry and Laboratory Medicine (IFCC), as the sample size exceeded the required number for nonparametric estimation [41]. This sample size falls within the range considered acceptable for nonparametric reference interval estimation according to CLSI C28-A3 guidelines. Reference intervals (RIs) were established using data obtained from 88 healthy cats with Reference Value Advisor v.2.0 implemented in a Microsoft Excel environment. Because nonparametric methods were used, no assumptions regarding data distribution were required. In addition, 90% confidence intervals (CIs) for the reference limits and 95% reference intervals were calculated in accordance with the 2012 guidelines of the American Society for Veterinary Clinical Pathology (ASVCP) [42]. The width of the 90% confidence intervals was evaluated following IFCC–CLSI recommendations. Statistical analyses for each subgroup were performed using SPSS version 27. The Shapiro–Wilk test was used to assess the normality assumption for each group. Outlier detection was reassessed using an objective statistical approach, namely Horn’s algorithm combined with Tukey’s interquartile fences (1.5 × IQR) based on Tukey’s hinges, in accordance with CLSI EP28-A3c and ASVCP recommendations. No statistically significant outliers were identified; therefore, no observations were excluded from the analysis. As the data were not normally distributed, intergroup comparisons were performed using the Mann–Whitney U test. Statistical significance was defined as *p* < 0.05.

## 3. Results

### 3.1. Study Population

The analyzed sample comprised 88 clinically normal mixed-breed cats, of which 33 were male (37.5%) and 55 were female (62.5%). A median age of 3 years was recorded among the enrolled cats. The characteristics of the study population are outlined in Table 1.

### 3.2. Complete Blood Count Parameters and Hematological Ratios

**Figure 1:**
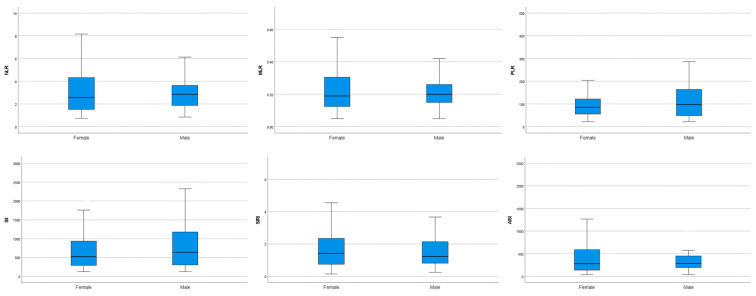
Box-plots of each ratio comparing females and males. Box-plots are presented for descriptive visualization of data distribution and sex-related differences; outlier identification was performed using objective statistical methods as described in the methods section. Abbreviations: NLR, neutrophil-to-lymphocyte ratio; MLR, monocyte-to-lymphocyte ratio; PLR, platelet-to-lymphocyte ratio; SII, systemic immune–inflammatory index; SIRI, systemic inflammatory response index; AISI, aggregate systemic inflammation index.

The mean ± standard deviation values of the CBC parameters are presented in Table 2. Following objective outlier assessment, no statistically significant outliers were identified for any inflammatory index in either sex; therefore, all observations were retained for analysis. Reference intervals for the hematological indices were established as follows: 0.8–7.1 for NLR; 0.1–0.6 for MLR; 22.7–374.6 for PLR; 130–2454 for SII; 39.5–1542.8 for AISI; and 0.2–6.1 for SIRI (Table 3). Comparison of male and female cats showed no statistically significant variation in NLR, MLR, PLR, SII, AISI, or SIRI (Table 4, Figure 1).

## 4. Discussion

In this retrospective study, reference intervals for hemogram-derived inflammatory markers (NLR, MLR, PLR, SII, AISI, and SIRI) were established in a healthy cat population, and no significant differences were observed between sexes for these markers. Previous studies in cats have investigated hematological indices in various inflammatory conditions and compared these indices with those from relatively small populations of healthy cats.

In the present study, the median value of NLR was 2.6, with minimum and maximum values of 0.8 and 7.1, respectively. Previously reported median NLR values in healthy cats range between 1.7 and 2.49, with the upper limit reaching as high as 40; however, the upper reference limit in our study (7.1) was lower, which may be attributed to the age of the cats included, possibly due to an age-related decline in lymphocyte counts [4,33,43,44,45,46,47,48]. Although increases in NLR have been documented in inflammatory and neoplastic disorders in cats, endogenous cortisol and catecholamines can rise during physiological stress and fear, leading to an increase in neutrophil counts and a decrease in lymphocyte counts. Therefore, any cause of physiological stress may lead to an increase in NLR [49]. Stress is common among cats, and numerous physiological and pathological conditions can elicit stress responses. The stress response system encompasses behavioral, physiological, and immunological reactions [50]. An early increase in circulating mature neutrophils occurs mainly through demargination. This phenomenon holds particular importance in feline species, as the marginal neutrophil compartment constitutes a larger fraction of the total neutrophil pool relative to other animals [51].

In the present study, the median MLR was 0.2, with minimum and maximum values of 0.1–0.6, respectively. Previously reported median MLR values in healthy cats range between 0.095 and 0.14, with upper limits generally below 0.5 [3,33,46,47]. Although minor differences exist among studies, these variations likely reflect population characteristics, inclusion criteria, and methodological differences in reference interval determination rather than true biological discrepancies. Monocytosis can occur whenever neutrophilia develops due to a shared bipotential stem cell. This phenomenon may also result from both stress and the mobilization of marginal cells within blood vessels due to elevated endogenous or exogenous cortisol levels, and it has been observed in cats [52]. The differing reference intervals reported in the studies may be attributable to this phenomenon.

In the present study, the median PLR was 91, with minimum and maximum values of 22.7 and 374.6, respectively. In a study comparing PLR in cats with chronic kidney disease to 32 healthy cats, the median PLR in the healthy cats was 123.3, with minimum and maximum values of 11.3 and 666.7, respectively [33]. In another study comparing PLR in cats with inflammation to 65 healthy cats, the maximum PLR in the healthy cat group was 528.3, which was suggested as a potential threshold for cats without inflammation [46]. The PLR values reported in our study are consistent with those in previous studies. Stress-induced increases in cortisol can elevate platelet counts, leading to the release of platelets into peripheral blood and transient lymphopenia [53]; consequently, the observed variations in PLR reference intervals may be influenced by this phenomenon. Differences have been observed in the reported NLR, MLR, and PLR values in healthy cats used as small control groups in the literature. These variations may be attributable to factors such as the type of hematology analyzer, differing statistical methods, population characteristics, and variations in the definition of healthy animals, potentially contributing to discrepancies between studies. Taken together, differences among published feline reference intervals should not be interpreted solely as consequences of age, breed, or analytical variation. Instead, they likely reflect the dynamic, stress-responsive, and immunologically sensitive characteristics of feline hematology, particularly when expressed through composite inflammatory ratios. Establishing reference intervals within clearly defined healthy populations remains essential to improve their interpretability in clinical decision making.

In the present study, the median SII value was 575.9, with minimum and maximum values of 130 and 2454, respectively. Previously reported median SII values in healthy cats have ranged between 259 and 431, with the upper limit reaching as high as 6.433 [3,33,34]. This finding may be attributed to factors such as body condition. Obesity has been shown to increase the SII in association with a heightened inflammatory state [54]. Body condition was not considered as a variable in the present study. The width of the reference range reported in that study yielded comparable results to those of the current study. In the present study, the median AISI value was 281.4, with minimum and maximum values of 39.5 and 1542.8, respectively. The median SIRI value was 1.3, with minimum and maximum values of 0.2 and 6.1, respectively. Data on AISI and SIRI levels in cats are quite limited in the current literature. In a single study, the median AISI value was 607.43 in cats with arterial thromboembolism, whereas in the same study the median AISI value in 10 healthy cats was 73.42 [3]. In cats with cardiomyopathy accompanied by cardiogenic pulmonary edema and cardiogenic pleural effusion, SIRI values were found to be significantly higher than those in healthy cats. In cats with pulmonary edema, the median SIRI value was 0.995, with minimum and maximum values of 0.380 and 3.035, respectively, whereas in cats with pleural effusion, the median SIRI value was 2.11, with minimum and maximum values of 0.42 and 3.31, respectively. The mean age of the cats included in that study was reported to be 13 years [4]. In cats with arterial thromboembolism, the median SIRI value was 2.28, whereas in the same study the median SIRI value in 10 healthy cats was 0.28 [3]. Importantly, indices such as SII, AISI, and SIRI mathematically combine three or more variables. From a statistical standpoint, the propagation of variability across multiple components inherently broadens reference intervals. Therefore, wider ranges observed in some studies do not necessarily indicate instability or methodological weakness but may reflect the composite biological nature of these indices.

The retrospective design of this study inherently introduced certain limitations. Most notably, blood smear analyses were not performed, limiting the ability to detect platelet aggregates and validate differential leukocyte measurements. Furthermore, potentially influential variables, including reproductive status and body condition, were not evaluated. In addition, the lack of PCR testing to exclude subclinical diseases (e.g., FeLV/FIV) was another limitation. Biochemical profiling was also not performed, which may have limited the ability to exclude subclinical systemic disorders. Furthermore, the classification of cats as clinically healthy was based on physical examination findings and routine laboratory results, which may not completely exclude subclinical or early stage diseases. Although urinalysis (urine dipstick testing) was performed in all cats, microscopic urine sediment examination was not available. Therefore, subclinical urinary abnormalities could not be completely excluded, which represents a limitation of the present study. Additionally, all cats were sourced from the same shelter, resulting in relatively homogeneous environmental, dietary, and stress conditions. While this homogeneity may reduce biological variability, it may also limit the generalizability of the findings to the broader feline population. Due to the limited age range of the study population (1–6 years), age-related comparisons could not be performed, which represents a limitation of the present study. Another limitation is that the sample size of 88, while within the acceptable range for nonparametric reference interval estimation according to CLSI C28-A3 guidelines, is below the ideal threshold of 120 individuals; this may have contributed to the relatively wide 90% confidence intervals observed for certain parameters. Finally, as the vaccination history of the cats was unknown, the observed immunological differences may also have affected the results.

## 5. Conclusions

This study is the first to provide reference intervals for hematological indices in clinically healthy cats. The established reference intervals were 0.8–7.1 for NLR, 0.1–0.6 for MLR, 22.7–374.6 for PLR, 130–2454 for SII, 39.5–1542.8 for AISI, and 0.2–6.1 for SIRI. These calculated biomarkers may have clinical relevance for specific pathologies and are useful in practice because they are easily measurable, inexpensive, and readily accessible parameters. These hematological parameters provide meaningful diagnostic insight at no additional cost to owners and can potentially minimize the blood volume required for further testing, an important benefit in feline patients. Future studies conducted in cats with known body condition, vaccination history, parasite control status, and reproductive status should further refine these intervals. In addition, investigating their role in detecting subclinical chronic inflammation and establishing breed specific reference intervals may enhance their diagnostic utility.

## Figures and Tables

**Table 1:** Baseline characteristics of the study population.

Variable	Value
Total cats	88
Sex	33 males and 55 females
Median age	3 years

**Table 2:** Complete blood count parameters (mean ± SD) obtained from healthy cats.

	N	Mean ± SD
WBC (10^9^/L)	88	11.38 ± 3.53
LYM (10^9^/L)	88	2.92 ± 1.43
MON (10^9^/L)	88	0.57 ± 0.25
NEU (10^9^/L)	88	7.09 ± 2.70
RBC (10^12^/L)	88	10.01 ± 1.98
MCV (fL)	88	41.26 ± 5.26
MCHC (g/L)	88	348.19 ± 13.99
RDW (fL)	88	31.35 ± 5.41
Hb (g/dL)	88	14.20 ± 2.43
HCT (%)	88	41 ± 6.8
PLT (10^9^/L)	88	241.19 ± 104.44

Abbreviations: n, number of data included in the statistical analysis; WBC, total white blood cell; LYM, lymphocyte; MON, monocyte; NEU, neutrophil; RBC, red blood cell; MCV, mean corpuscular volume; MCHC, mean corpuscular hemoglobin concentration; RDW, red blood cell distribution width; Hb, hemoglobin; HCT, hematocrit; PLT, platelet count.

**Table 3:** Reference intervals for hemogram-derived inflammatory markers in healthy cats.

Analyte	n	Median	Mean ± SD	RI	IQR	LRL (90%)	URL (90%)	Method
NLR (10^9^/L)	88	2.6	2.96 ± 1.68	0.8–7.1	2.18	0.7–1.0	6.1–8.2	NP
MLR (10^9^/L)	88	0.2	0.23 ± 0.12	0.1–0.6	0.16	0.0–0.1	0.5–0.6	NP
PLR (10^9^/L)	88	91.0	105.42 ± 77.71	22.7–374.6	71.02	22.3–30.7	254.5–443.4	NP
SII (10^9^/L)	88	575.9	737.13 ± 570.43	130–2454	672.75	122.7–149.6	1894.8–2664.7	NP
AISI (10^9^/L)	88	281.4	427.72 ± 399.87	39.5–1542.8	431.05	35.8–64.4	1237.2–2200.5	NP
SIRI (10^9^/L)	88	1.3	1.76 ± 1.42	0.2–6.1	1.58	0.1–0.4	4.8–6.5	NP

Abbreviations: n, number of data included in the statistical analysis; RI, reference interval; IQR, interquartile range; LRL, lower reference limit; URL, upper reference limit; NP, nonparametric; NLR, neutrophil-to-lymphocyte ratio; MLR, monocyte-to-lymphocyte ratio; PLR, platelet-to-lymphocyte ratio; SII, systemic immune–inflammatory index; AISI, aggregate systemic inflammation index; SIRI, systemic inflammatory response index.

**Table 4:** Comparison of the different inflammatory ratios between females and males.

Analyte	n	95% CI for Mean	Female Mean ± SD	Female Median (25th and 75th Percentiles)	Male Mean ± SD	Male Median (25th and 75th Percentiles)	Cohen’s d	*p*-Value
NLR (10^9^/L)	55 (F) 33 (M)	2.48–3.42 2.43–3.56	2.95 ± 1.74	2.58 (1.53–4.33)	2.99 ± 1.60	2.86 (1.88–2.86)	−0.026	0.727
MLR (10^9^/L)	55 (F) 33 (M)	0.20–0.27 0.18–0.26	0.23 ± 0.13	0.19 (0.13–0.31)	0.22 ± 0.11	0.20 (0.15–0.26)	0.083	0.907
PLR (10^9^/L)	55 (F) 33 (M)	78.12–105.20 90.64–166.08	91.66 ± 50.08	85.85 (55.67–121.92)	128.36 ± 106.39	97.81 (48.94–164.10)	−0.420	0.332
SII (10^9^/L)	55 (F) 33 (M)	548.51–796.14 591.56–1098.71	672.33 ± 458.01	521.060 (290.45–933.64)	845.13 ± 715.13	638.46 (303.21–1176.62)	−0.266	0.549
AISI (10^9^/L)	55 (F) 33 (M)	323.53–549.13 280.44–546.33	436.33 ± 417.26	278.04 (136.69–589.17)	413.38 ± 374.93	284.81 (193.53–449.87)	0.055	0.997
SIRI (10^9^/L)	55 (F) 33 (M)	1.46–2.32 1.17–1.92	1.89 ± 1.60	1.42 (0.75–2.35)	1.54 ± 1.06	1.23 (0.81–2.14)	0.230	0.651

Abbreviations: 95% CI, %95 confidence interval; SD, standard deviation; NLR, neutrophil-to-lymphocyte ratio; MLR, monocyte-to-lymphocyte ratio; PLR, platelet-to-lymphocyte ratio; SII, systemic immune–inflammatory index; AISI, aggregate systemic inflammation index; SIRI, systemic inflammatory response index; F, female; M, male. Cohen’s d shows the standardized difference between group means; negative values indicate higher male means. *p* < 0.05 is considered statistically significant.

## Data Availability

The original contributions presented in this study are included in the article. Further inquiries can be directed to the corresponding author.

## References

[1] Tharangani R.W.P., Skerrett-Byrne D.A., Gibb Z., Nixon B., Swegen A. (2022). The future of biomarkers in veterinary medicine: Emerging approaches and associated challenges. Animals.

[2] Erdogan H., Ozalp T., Erdogan S., Ural K. (2025). Assessment of novel haematological inflammatory markers (NLR, SII, and SIRI) as predictors of sirs in dogs with canine monocytic ehrlichiosis. Vet. Stanica.

[3] Esin C., Uzun B. (2025). Prognostic and diagnostic value of systemic inflammatory blood markers (NLR, MLR, PLR, AISI, SIRI, and SII) in feline arterial thromboembolism. Vet. Immunol. Immunopathol..

[4] Krofič Žel M., Song K.H., Nemec Svete A., Domanjko Petrič A. (2025). Evidence for chronic inflammation in cats with cardiomyopathies. J. Feline Med. Surg..

[5] Sevim K., Çolakoğlu E.Ç., Kaya U. (2025). Evaluation of hematologic indices in parvovirus infected dogs with systemic inflammatory response syndrome (SIRS). Top. Companion Anim. Med..

[6] Petrucci G.N., Lobo L., Queiroga F., Martins J., Prada J., Pires I., Henriques J. (2021). Neutrophil-to-lymphocyte ratio is an independent prognostic marker for feline mammary carcinomas. Vet. Comp. Oncol..

[7] Jin N., Huang L., Hong J., Zhao X., Hu J., Wang S., Chen X., Rong J., Lu Y. (2023). The association between systemic inflammation markers and the prevalence of hypertension. BMC Cardiovasc. Disord..

[8] Xia Y., Xia C., Wu L., Li Z., Li H., Zhang J. (2023). Systemic immune inflammation index (SII), system inflammation response index (SIRI) and risk of all-cause mortality and cardiovascular mortality: A 20-year follow-up cohort study of 42,875 US adults. J. Clin. Med..

[9] Balta S., Ozturk C. (2015). The platelet-lymphocyte ratio: A simple, inexpensive and rapid prognostic marker for cardiovascular events. Platelets.

[10] Pierini A., Gori E., Lippi I., Ceccherini G., Lubas G., Marchetti V. (2019). Neutrophil-to-lymphocyte ratio, nucleated red blood cells and erythrocyte abnormalities in canine systemic inflammatory response syndrome. Res. Vet. Sci..

[11] Neumann S. (2021). Neutrophil-to-lymphocyte and platelet-to-lymphocyte ratios in dogs and cats with acute pancreatitis. Vet. Clin. Pathol..

[12] Liu Z., Li Y., Wang Y., Zhang H., Lian Y., Cheng X. (2022). The neutrophil-to-lymphocyte and monocyte-to-lymphocyte ratios are independently associated with the severity of autoimmune encephalitis. Front. Immunol..

[13] González-Domínguez A., Cristobal-Verdejo J.I., López-Espinar C., Fontela-González S., Vázquez S., Justo-Domínguez J., González-Caramazana J., Bragado Cuesta M., Álvarez-Punzano A., Herrería-Bustillo V.J. (2024). Retrospective evaluation of hematological ratios in canine parvovirosis: 401 cases. J. Vet. Intern. Med..

[14] Hodgson N., Llewellyn E.A., Schaeffer D.J. (2018). Utility and prognostic significance of neutrophil-to-lymphocyte ratio in dogs with septic peritonitis. J. Am. Anim. Hosp. Assoc..

[15] Dinler Ay C. (2022). Neutrophil to lymphocyte ratio as a prognostic biomarker in puppies with acute diarrhea. J. Vet. Emerg. Crit. Care.

[16] Johnson M.M., Gicking J.C., Keys D.A. (2023). Evaluation of red blood cell distribution width, neutrophil-to-lymphocyte ratio, and other hematologic parameters in canine acute pancreatitis. J. Vet. Emerg. Crit. Care.

[17] Sottnik J.L., Rao S., Lafferty M.H., Thamm D.H., Morley P.S., Withrow S.J., Dow S.W. (2010). Association of blood monocyte and lymphocyte count and disease-free interval in dogs with osteosarcoma. J. Vet. Intern. Med..

[18] Marconato L., Martini V., Stefanello D., Moretti P., Ferrari R., Comazzi S., Laganga P., Riondato F., Aresu L. (2015). Peripheral blood lymphocyte/monocyte ratio as a useful prognostic factor in dogs with diffuse large B-cell lymphoma receiving chemoimmunotherapy. Vet. J..

[19] Skor O., Fuchs-Baumgartinger A., Tichy A., Kleiter M., Schwendenwein I. (2017). Pretreatment leukocyte ratios and concentrations as predictors of outcome in dogs with cutaneous mast cell tumours. Vet. Comp. Oncol..

[20] Marcos R., Oliveira J., Jorge R., Marcos C., Rosales C. (2023). Neutrophil to lymphocyte ratio and principal component analysis offer prognostic advantage for dogs with mammary tumors. Front. Vet. Sci..

[21] Benvenuti E., Pierini A., Gori E., Lucarelli C., Lubas G., Marchetti V. (2020). Neutrophil-to-lymphocyte ratio (NLR) in canine inflammatory bowel disease (IBD). Vet. Sci..

[22] Becher A., Suchodolski J.S., Steiner J.M., Heilmann R.M. (2021). Blood neutrophil-to-lymphocyte ratio (NLR) as a diagnostic marker in dogs with chronic enteropathy. J. Vet. Diagn. Investig..

[23] Cristóbal J.I., Duque F.J., Usón-Casaús J., Barrera R., López E., Pérez-Merino E.M. (2022). Complete blood count-derived inflammatory markers changes in dogs with chronic inflammatory enteropathy treated with adipose-derived mesenchymal stem cells. Animals.

[24] DeProspero D.J., Hess R.S., Silverstein D.C. (2023). Neutrophil-to-lymphocyte ratio is increased in dogs with acute congestive heart failure secondary to myxomatous mitral valve disease compared to both dogs with heart murmurs and healthy controls. J. Am. Vet. Med. Assoc..

[25] Filipe S., Dall’Ara P., Razzuoli E., Ciliberti M.G., Becher A., Acke E., Serrano G., Kiefer I., Alef M., Von Bomhard W. (2024). Evaluation of the blood neutrophil-to-lymphocyte ratio (NLR) as a diagnostic and prognostic biomarker in dogs with portosystemic shunt. Vet. Sci..

[26] Tuna G.E. (2024). Neutrophil-to-lymphocyte ratio and platelet-to-lymphocyte ratio in dogs with various degrees of myxomatous mitral valve disease. Egypt. J. Vet. Sci..

[27] Chiti L.E., Martano M., Ferrari R., Boracchi P., Giordano A., Grieco V., Buracco P., Iussich S., Giudice C., Miniscalco B. (2020). Evaluation of leukocyte counts and neutrophil-to-lymphocyte ratio as predictors of local recurrence of feline injection site sarcoma after curative intent surgery. Vet. Comp. Oncol..

[28] Tagawa M., Shimbo G., Miyahara K. (2021). Prognostic role of lymphocyte to monocyte ratio in feline high-grade lymphomas. Can. Vet. J..

[29] Cagnasso F., Bruno B., Zanatta R., Bellino C., Roncone S., Borella F., Gianella P., Borrelli A. A retrospective evaluation of neutrophil–lymphocyte ratio, monocyte–lymphocyte ratio and platelet–lymphocyte ratio in cats with obstructive uropathy. Proceedings of the 75th Convegno SISVet.

[30] Cao Y., Li P., Zhang Y., Qiu M., Li J., Ma S., Yan Y., Li Y., Han Y. (2023). Association of systemic immune inflammatory index with all-cause and cause-specific mortality in hypertensive individuals: Results from NHANES. Front. Immunol..

[31] Wang H., Nie H., Bu G., Tong X., Bai X. (2023). Systemic immune-inflammation index (SII) and the risk of all-cause, cardiovascular, and cardio-cerebrovascular mortality in the general population. Eur. J. Med. Res..

[32] Luo Y., Yang L., Cheng X., Bai Y., Xiao Z. (2025). The association between blood count based inflammatory markers and the risk of atrial fibrillation, heart failure and cardiovascular mortality. Sci. Rep..

[33] Krofič Žel M., Nemec Svete A., Tozon N., Pavlin D. (2024). Hemogram-derived inflammatory markers in cats with chronic kidney disease. Animals.

[34] Aydın Ö. (2025). Investigation of novel hematological index variations in cats naturally infected with feline panleukopenia virus. Kocatepe Vet. J..

[35] Tršar L., Štrljič M., Nemec Svete A., Koprivec S., Tozon N., Krofič Žel M., Pavlin D. (2025). Evaluation of selected inflammatory markers in cats with feline infectious peritonitis before and after therapy. BMC Vet. Res..

[36] Fois A.G., Paliogiannis P., Scano V., Cau S., Babudieri S., Perra R., Ruzzittu G., Zinellu E., Pirina P., Carru C. (2020). The systemic inflammation index on admission predicts in-hospital mortality in COVID-19 patients. Molecules.

[37] Ghobadi H., Mohammadshahi J., Javaheri N., Fouladi N., Mirzazadeh Y., Aslani M.R. (2022). Role of leukocytes and systemic inflammation indexes (NLR, PLR, MLP, dNLR, NLPR, AISI, SIR-I, and SII) on admission predicts in-hospital mortality in non-elderly and elderly COVID-19 patients. Front. Med..

[38] Zinellu A., Collu C., Nasser M., Paliogiannis P., Mellino S., Zinellu E., Traclet J., Ahmad K., Mangoni A.A., Carru C. (2021). The aggregate index of systemic inflammation (AISI): A novel prognostic biomarker in idiopathic pulmonary fibrosis. J. Clin. Med..

[39] Hrubaru I., Motoc A., Moise M.L., Miutescu B., Citu I.M., Pingilati R.A., Popescu D.E., Dumitru C., Gorun F., Olaru F. (2022). The predictive role of maternal biological markers and inflammatory scores NLR, PLR, MLR, SII, and SIRI for the risk of preterm delivery. J. Clin. Med..

[40] Hosseninia S., Ghobadi H., Garjani K., Hosseini S.A.H., Aslani M.R. (2023). Aggregate index of systemic inflammation (AISI) in admission as a reliable predictor of mortality in COPD patients with COVID-19. BMC Pulm. Med..

[41] Geffré A., Concordet D., Braun J.P., Trumel C. (2011). Reference value advisor: A new freeware set of macroinstructions to calculate reference intervals with Microsoft Excel. Vet. Clin. Pathol..

[42] Friedrichs K.R., Harr K.E., Freeman K.P., Szladovits B., Walton R.M., Barnhart K.F., Blanco-Chavez J. (2012). ASVCP reference interval guidelines: Determination of de novo reference intervals in veterinary species and other related topics. Vet. Clin. Pathol..

[43] Konstantinidis A.O., Adamama-Moraitou K.K., Griggs A., Musser M.L., Nenninger A.S., Soubasis N., Pardali D., Mylonakis M.E., Jergens A.E. (2025). Blood leukocyte ratios as predictive markers of chronic enteropathy phenotypes in cats. Vet. Sci..

[44] Heaton P.R., Blount D.G., Mann S.J., Devlin P., Koelsch S., Smith B.H.E., Stevenson J., Harper E.J., Rawlings J.M. (2002). Assessing age-related changes in peripheral blood leukocyte phenotypes in domestic shorthaired cats using flow cytometry. J. Nutr..

[45] Campbell D.J., Rawlings J.M., Koelsch S., Wallace J., Strain J.J., Hannigan B.M. (2004). Age-related differences in parameters of feline immune status. Vet. Immunol. Immunopathol..

[46] Donato G., Pennisi M.G., Persichetti M.F., Archer J., Masucci M. (2023). A retrospective comparative evaluation of selected blood cell ratios, acute phase proteins, and leukocyte changes suggestive of inflammation in cats. Animals.

[47] Rossi A., Proverbio D., Perego R., Baggiani L., Spada E. (2024). Evaluation of leukocyte ratios as survival prognostic markers in feline retrovirus infections. Vet. J..

[48] Gori E., Pierini A., Lippi I., Lubas G., Marchetti V. (2021). Leukocytes ratios in feline systemic inflammatory response syndrome and sepsis: A retrospective analysis of 209 cases. Animals.

[49] Fam A.L.P.D., Rocha R.M.V.M., Pimpão C.T., Cruz M.A. (2010). Alterations on leukogram of domestic felines (Felis catus) due to acute and chronic stress. Rev. Acad. Ciênc. Agrár. Ambient..

[50] Stella J., Croney C., Buffington T. (2013). Effects of stressors on the behavior and physiology of domestic cats. Appl. Anim. Behav. Sci..

[51] Valenciano A.C., Decker L.S., Cowell R.L., Weiss D.J., Wardrop K.J. (2010). Interpretation of feline leukocyte responses. Schalm’s Veterinary Hematology.

[52] Latimer K., Prasse K.W., Latimer K., Mahaffey E.A., Prasse K.W. (2003). Leukocytes. Duncan & Prasse’s Veterinary Laboratory Medicine: Clinical Pathology.

[53] Pierini A., Esposito G., Gori E., Benvenuti E., Ruggiero P., Lubas G., Marchetti V. (2021). Platelet abnormalities and platelet-to-lymphocyte ratios in canine immunosuppressant-responsive and non-responsive enteropathy: A retrospective study in 41 dogs. J. Vet. Med. Sci..

[54] Tuna G.E., Ulutaş B. (2024). The systemic-immune inflammatory index in naturally obese dogs. Assiut Vet. Med. J..

